# Measurement and Analysis of Magnetic Properties of Permalloy for Magnetic Shielding Devices under Different Temperature Environments

**DOI:** 10.3390/ma16083253

**Published:** 2023-04-20

**Authors:** Jinji Sun, Jianyi Ren, Jin Li, Yuejing Huang

**Affiliations:** 1 Key Laboratory of Ultra-Weak Magnetic Field Measurement Technology, Ministry of Education, School of Instrumentation and Optoelectronic Engineering, Beihang University, Beijing 100191, China; sunjinji@buaa.edu.cn (J.S.); renjianiy@buaa.edu.cn (J.R.); huangyj99@buaa.edu.cn (Y.H.); 2 Zhejiang Provincial Key Laboratory of Ultra-Weak Magnetic-Field Space and Applied Technology, Hangzhou Innovation Institute, Beihang University, Hangzhou 310052, China

**Keywords:** magnetic materials, permalloy, temperature dependence, magnetic properties, magnetic shielding devices

## Abstract

The relative permeability, coercivity, and remanence of permalloy are closely related to the performance of magnetic shielding devices. In this paper, the relationship between the magnetic properties of permalloy and the working temperature of magnetic shielding devices is measured. Firstly, the measurement method of permalloy properties based on the simulated impact method is analyzed. What is more, a magnetic property test system consisting of a soft magnetic material tester and a high–low temperature chamber for permalloy ring samples at different temperatures was established to measure DC and AC (0.01 Hz to 1 kHz) magnetic properties at different temperatures (−60 °C to 140 °C). Finally, the results show that compared with room temperature (25 °C), the initial permeability ($\mu_{i}$) decreases by 69.64% at −60 °C and increases by 38.23% at 140 °C, and the coercivity ($h_{c}$) decreases by 34.81% at −60 °C and increases by 8.93% at 140 °C, which are the key parameters in the magnetic shielding device. It can be concluded that the relative permeability and remanence of permalloy are positively correlated with temperature, while the saturation magnetic flux density and coercivity are negatively correlated with temperature. This paper is of great significance to the magnetic analysis and design of magnetic shielding devices.

## 1. Introduction

With the increasing requirements for ultra-low magnetic fields applied in biomedical signal processing [1], fundamental physic experiments [2], and aerospace engineering [3], the application of magnetic shielding devices is becoming increasingly widespread. When detecting the magnetic signal generated by the neural current to diagram the magnetocardiography and magnetoencephalography [4], measuring the electric dipole moment (EDM) of basic particles [5,6], and achieving the sensitive property of a spin-exchange relaxation free (SERF) atomic magnetometer [7,8,9], an ultra-low magnetic environment is essential, which usually requires reaching the nT level, where weak magnetic target signals can be rarely disturbed by the surrounding magnetic field.

In magnetic shielding devices, the attenuation of the external magnetic field by the passive shielding layer is achieved by using high permeability material to shunt magnetic flux [10,11], which means that the magnetic properties of the passive shielding layer material will limit the magnetic shielding effect. At the same time, the remanence and coercivity of the passive shielding layer also affect the residual magnetic field inside the magnetic shielding device. The magnetic properties of magnetic materials are extremely susceptible to external environmental influences, such as temperature, stress, etc. [12,13,14,15], which in turn affect the residual magnetic field inside the magnetic shielding device. For ultra-high sensitivity SERF atomic magnetometers, in order to achieve the atomic spin effect, the internal air chamber needs to be heated to a temperature of 150 °C [16,17]. Although some thermal insulation measures have been taken, the temperature of the inner magnetic shielding device will still be higher than room temperature. According to experimental records of magnetic shielding devices used in SERF devices, the temperature of the magnetic shielding layer near the air chamber is often between 40 °C and 50 °C. On the other hand, large magnetic shielding devices cannot provide constant temperature conditions, and external ambient temperatures that usually vary from −10 °C to 40 °C during a year [18] can also affect the magnetic properties of the passive shielding layer. Therefore, studying the effect of temperature on the magnetic properties of magnetic materials is of great significance for the magnetic field analysis and design of magnetic shielding devices. In order to obtain a complete relationship between the magnetic properties of permalloy and temperature, the temperature range considered in this paper is −60 °C to 140 °C.

Many scholars have analyzed the relationship between temperature and magnetic properties of magnetic materials, with the main purpose of analyzing the impact of different temperatures in electric motors on the permeability and loss of magnetic materials. Xiao et al. [19] conducted experiments to analyze the magnetic properties of silicon steel in a thermal stress coupled environment. The results show that relative permeability increases with increasing temperature, and there is an inverse relationship between permeability and pressure. Yang et al. [20] measured and analyzed the magnetic properties of ferrite, nanocrystalline, and amorphous alloys at different temperatures (25–120 °C) and high frequencies (1–20 kHz). The temperature dependence of three materials under different conditions was systematically compared using a loss coefficient of variation (CLV) in the experimental materials, and their respective suitable usage conditions were analyzed. Zhang et al. [21] measured the magnetic properties of amorphous/nanocrystalline powder cores, X-flux (Fe-Si), Sendust (Fe-Si-Al), and ferrite in the range of 20 to 100 kHz and discussed magnetic properties including temperature-dependent initial permeability, saturated magnetic flux density, and core loss. It has been proved that the magnetic properties are related to temperature and frequency, and the nanocrystalline powder core has relatively good temperature stability and relatively low loss. Dmitriy et al. [22] have studied the magnetic impedance and stress impedance effects of Amorphous CoFeSiB at different temperatures and have shown that high temperatures have a significant impact on magnetic impedance and stress impedance. Dafri et al. [23] tested ferrites under different thermal conditions and observed their magnetic properties, including coercivity, remanence, and saturation flux density. According to the results, these properties have been shown to be negatively correlated with temperature. Brialmont et al. [24] proved that by measuring Ni-5at.%. The relative permeability and peak strength of the relative permeability of the W alloy are almost unaffected within the test temperatures of 77 K, 195 K, 238 K, and 297 K, respectively. Ladjimi et al. [25] introduced a temperature-dependent parameter k in the traditional Jiles–Atherton (JA) model to describe the thermal effect of hysteresis, which is very consistent with the measurement results of ferrite materials. Li et al. [14] provided a model for the temperature dependence of the hysteresis of Fe-Si magnetic sheets by combining the JA model and the Preisach model. Chen et al. [26] established a core loss model and corresponding dynamic hysteresis model for non-oriented silicon steel samples, considering the effect of temperature on eddy current loss.

In the above research, the thermal effects of various soft magnetic materials have been studied, including silicon steel, ferrite, amorphous, and nanocrystalline alloys, while few studies have specifically investigated the relationship between temperature and permalloy. For the magnetic shielding device used in ultra-high precision atomic magnetometers, the shielding performance (residual magnetic field and uniformity) of the magnetic shielding device will be affected by temperature. In order to accurately analyze the magnetic performance of the magnetic shielding device affected by temperature, it is necessary to obtain the relationship between temperature and permalloy magnetic properties. Therefore, the effect of temperature on the properties of permalloy was studied, and 1J85 (a typical permalloy produced in China, with about 80% Ni, 5% Mo, and iron) was used as the experimental object in this paper, because this is the most commonly used magnetic shielding material in DC and low-frequency (DC to 1 kHz) magnetic shielding devices. Firstly, the measurement methods and theories of magnetic properties of magnetic materials were discussed. Then, a measurement system was established to measure the magnetic properties of permalloy at different temperatures, and the DC and AC (0.01 Hz to 1 kHz) magnetic properties of permalloy were measured and analyzed. This paper measured and analyzed the correlation between the magnetic properties and temperature of permalloy, which is of great significance for the magnetic analysis and design of magnetic shielding devices.

The rest of this article is arranged as follows. In Section 2, the measurement and calculation principles of magnetic properties of permalloy are introduced first. Section 3 describes the measurement procedure and discusses the measurement results. Section 4 summarizes the conclusions.

## 2. Measuring Principle of Permalloy’s Magnetic Properties

### 2.1. Dimension Parameters of Permalloy

**Figure 1:**
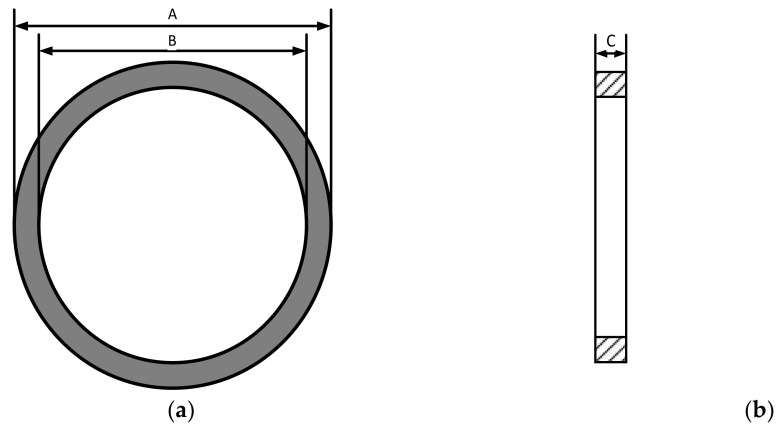
Schematic diagram of the size of the sample ring. (**a**) front view of the sample ring, (**b**) section view of the sample ring.

Magnetic materials are generally measured using the sample ring method, as shown in Figure 1.

In this paper, the effective magnetic circuit length $L_{e}$ and effective cross-sectional area $A_{e}$ of the sample ring are calculated according to the Chinese industry standard SJ/T10281:

(1) $$L_{e}=\frac{c_{1}^{2}}{c_{2}}$$

(2) $$A_{e}=\frac{c_{1}}{c_{2}}\times \frac{S_{x}}{100}$$

where $c_{1}$ and $c_{2}$ are the constant of the magnetic core and $\;S_{x}$ is the lamination coefficient.

(3) $$c_{1}=\frac{2\pi }{C\ln\frac{A}{B}}$$

(4) $$c_{2}=\frac{2\pi \left(\frac{2}{B}-\frac{2}{A}\right)}{C^{2}\ln^{3}\frac{A}{B}}$$

where *A* is the outer diameter, *B* is the inner diameter, and *C* is thickness of the sample ring.

### 2.2. Measuring of Permalloy’s Magnetic Properties

In this paper, the simulated impact method is used to measure the magnetization curve, hysteresis loop, and magnetic properties of magnetic materials. During the process of measurement, the object being measured is a sample ring, the excitation winding provides magnetic field excitation, while the induction winding induces the magnetic field generated by the sample ring.

Based on the Ampere circuit theorem, the excitation winding current I is used to generate the magnetic field strength H (A/m), which is expressed as:

(5) $$H=\frac{N_{1}I}{L_{e}}$$

where H is the magnetic field strength (A/m), $N_{1}$ is the turn number of the excitation winding, *I* is the excitation winding current (A), and $L_{e}$ is the effective magnetic path length of the sample ring (m).

For testing DC magnetic characteristics, the magnetic flux density B is calculated by inducing magnetic flux, so magnetic flux density during DC measurement is calculated as below:

(6) $$B=\frac{\varphi }{N_{2}A_{e}}$$

where B is the magnetic flux density (T), $N_{2}$ is the turn number of the induction winding, *φ* is the induced magnetic flux (Wb), and $A_{e}$ is the effective cross-sectional area of the sample ring (m^2^).

When testing AC magnetic characteristics, the magnetic flux density is calculated using the induced voltage of the induction winding. Therefore, the calculation formula for the magnetic flux density during AC measurement is

(7) $$B=\frac{V_{rms}}{4.44\times fN_{2}A_{e}}$$

where $V_{rms}$ is the effective value of the induced voltage (V) and f is the frequency of the AC magnetic field (Hz).

When parameters are determined, the hysteresis loop can be calculated based on the current I and the measured magnetic flux or voltage. In order to ensure the accuracy of the measurement, the turns of the excitation coil and the induction coil need to be kept in an appropriate range. For the excitation coil, since the instrument’s current output range is 0.01 mA to 10 A, in order to ensure that the magnetic field H is within the measurable magnetic field range of the measuring instrument, after the sample ring size is determined, the number of turns of the excitation coil should be analyzed in combination with Equation (5). Similarly, the number of turns of an induction coil should be analyzed according to Equations (6) and (7) and the magnetic flux or voltage range.

According to the measured hysteresis loops, the magnetic property parameters of magnetic materials can be analyzed, including initial permeability ($\mu_{i}$), maximum permeability ($\mu_{m}$), AC amplitude permeability ($\mu_{a}$), coercivity ($H_{c}$), remanence ($B_{r}$), and saturation flux density ($B_{s}$).

## 3. Measurement Results and Discussion

### 3.1. Measurement of Magnetic Properties at Different Temperatures

**Figure 2:**
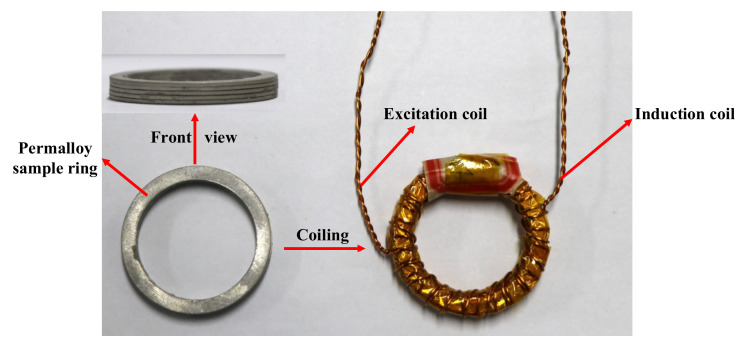
Schematic diagram of permalloy sample ring.

In this paper, a permalloy sample ring is used for magnetic property measurement. The sample ring is shown in Figure 2. Because magnetic shielding devices are mostly constructed using laminations, the sample ring is made of five 1 mm thick laminations, with dimensions shown in Table 1.

**Figure 3:**
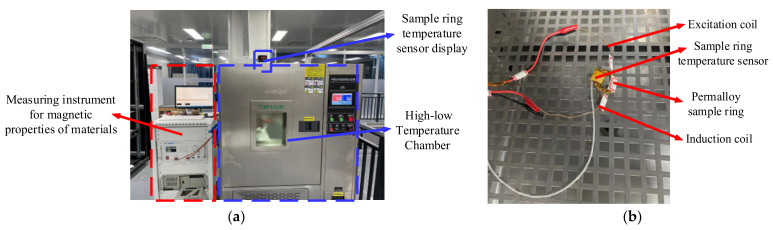
The magnetic property measurement system for permalloy at different temperatures (**a**) is external to the measurement system and (**b**) is internal to the high–low temperature chamber of the measurement system.

As shown in Figure 3, we put the permalloy into a high–low temperature chamber (temperature range: −70 °C to 150 °C, accuracy: 0.1 °C) to provide different temperature environments for the permalloy sample ring. We pasted a platinum resistor (PT100) on the sample surface to test the temperature of the sample ring. The magnetic properties of the sample ring at different ambient temperatures were measured using a material magnetic property measuring instrument (Hunan linkjoin Technology, China, MATS-3000S), including initial permeability ($\mu_{i}$), maximum permeability ($\mu_{m}$), AC amplitude permeability ($\mu_{a}$), coercivity ($H_{c}$), remanence ($B_{r}$), and saturation flux density ($B_{s}$). The measurement steps are as follows:

(1) Calculate the required number of excitation and induction coil turns based on the sample ring size information and perform winding;
(2) Place the wrapped sample ring into a high and low temperature box and affix a temperature sensor;
(3) Open the material magnetic property measuring instrument, preheat for 20 min, and connect the sample to the material magnetic property measuring instrument;
(4) Conduct a temperature rise experiment, and then change to a temperature drop experiment. When the temperature of the incubator reaches the specified temperature, wait for 20 min to observe whether the temperature of the sample temperature sensor is stable at the desired temperature. When stable, conduct a measurement;
(5) Measure the magnetization curve and hysteresis loop of the material at the set ambient temperature point, and then calculate magnetic properties such as permeability.

### 3.2. Analysis of Measurement Results

The magnetization curve and hysteresis loop at temperatures ranging from −60 °C to 140 °C are measured and the magnetic properties of the material are analyzed. Because of the demand for magnetic shielding devices, the emphasis is on the magnetic characteristics from −10 °C to 60 °C. In this range, the measuring step is 10 °C, and the rest steps are 20 °C.

**Figure 4:**
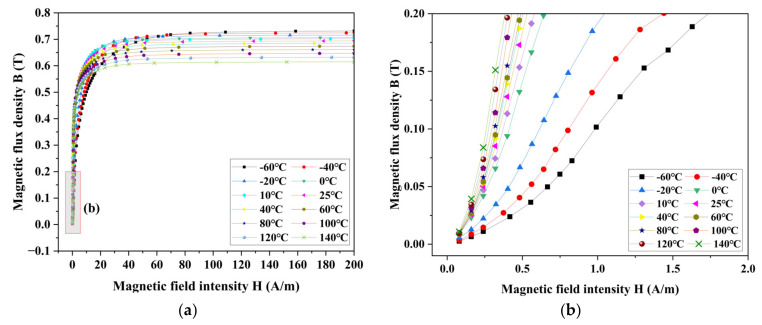
Measurement results of DC magnetization loop at different temperatures, (**a**) comparison of test results at −60 °C to 140 °C, and (**b**) initial portion after amplification.

Due to the low saturation magnetic field strength $H_{m}$ of permalloy, the maximum magnetic field strength set for testing in this article is 200 A/m. As shown in Figure 4, the initial slope of the permalloy magnetization curve increases, and the saturation flux density decreases.

**Figure 5:**
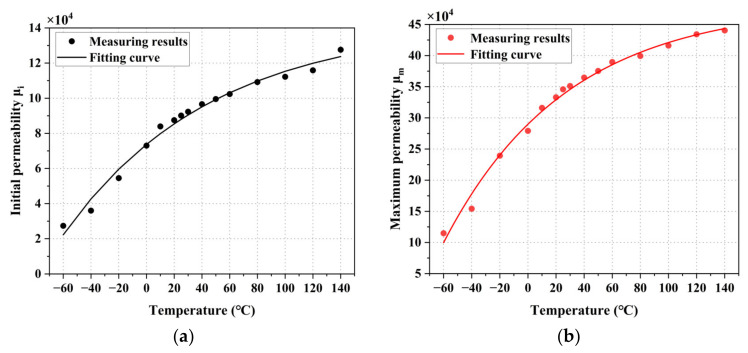
Measurement results of DC permeability at different temperatures, (**a**) initial permeability, and $\mu_{i}$ (**b**) maximum permeability $\mu_{m}$.

Based on the magnetization curve, the relationship between the initial permeability $\mu_{i}$ and maximum permeability $\mu_{m}$ of permalloy and temperature can be analyzed, as shown in Figure 5. According to the results, the initial permeability improves with temperature as an exponential function, and the maximum permeability also increases with temperature as an exponential function. Compared with room temperature (25 °C), the initial permeability decreases by 69.64% at −60 °C and the maximum permeability decreases by 38.23%. The initial permeability increases by 38.23% at 140 °C, and the maximum permeability decreases by 25.39%.

**Figure 6:**
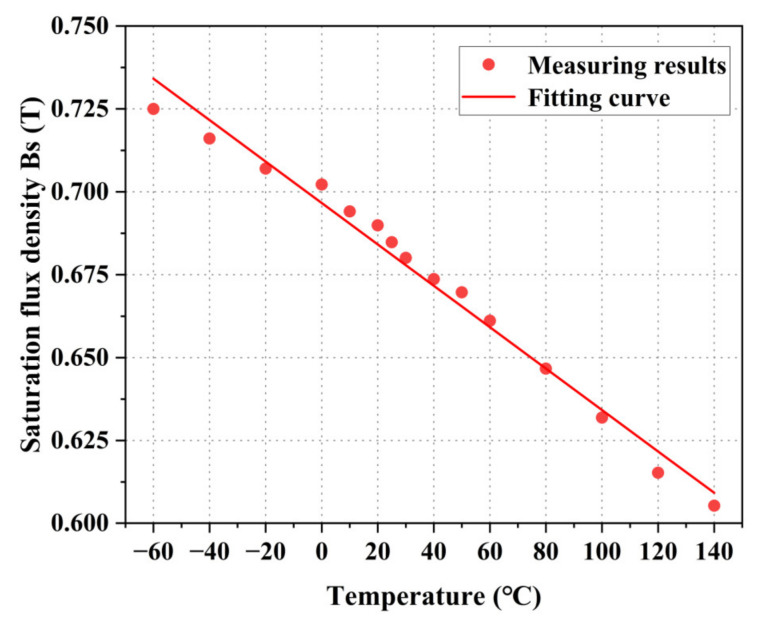
Measurement results of DC saturation flux density $B_{s}$ at different temperatures.

Based on the magnetization curve, the relationship between the saturation flux density $B_{s}$ of permalloy and temperature can be analyzed, as shown in Figure 6. According to the results, the saturation flux density decreases with the increase in temperature as a linear function. Compared to room temperature (25 °C), the saturation flux density increases by 5.87% at −60 °C and decreases by 11.01% at 140 °C.

**Figure 7:**
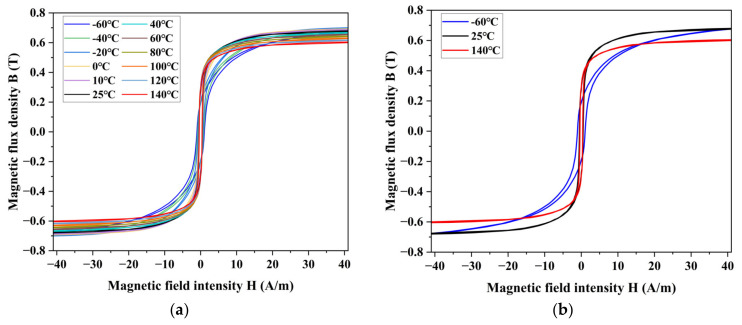
Measurement results of DC hysteresis loop at different temperatures, (**a**) comparison of test results at −60 °C to 140 °C, and (**b**) comparison of results at the highest, lowest, and room temperatures.

We measured the hysteresis loops of permalloy at different temperatures, as shown in Figure 7. According to the hysteresis loop, the parameters of the material can be analyzed, including coercivity ($H_{c}$), remanence ($B_{r}$), and saturation flux density ($B_{s}$).

**Figure 8:**
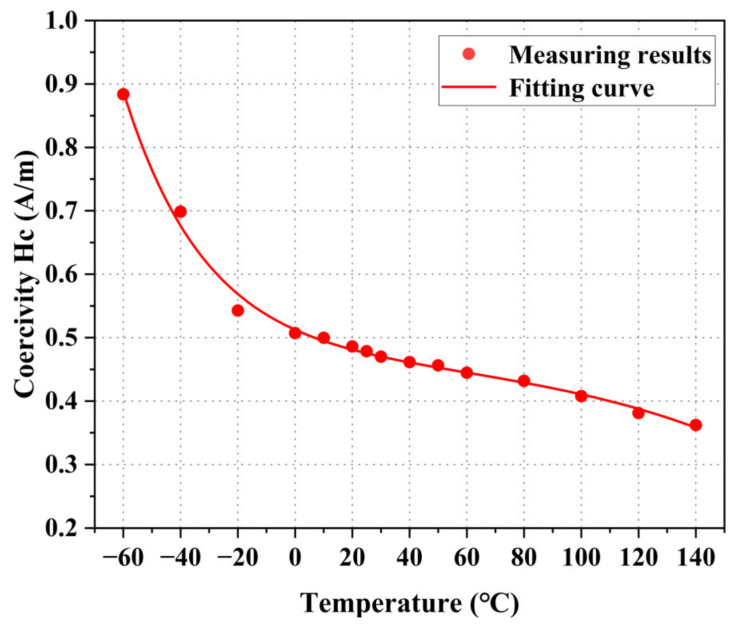
Measurement results of DC coercivity $H_{c}$ at different temperatures.

According to the hysteresis loop, the relationship between the coercivity $H_{c}$ and temperature of permalloy is analyzed, as shown in Figure 8. The results show that the coercivity recedes with increasing temperature as a function of the second-order exponential decay function. Compared with room temperature (25 °C), the coercivity increases by 84.64% at −60 °C and decreases by 22.91% at 140 °C.

**Figure 9:**
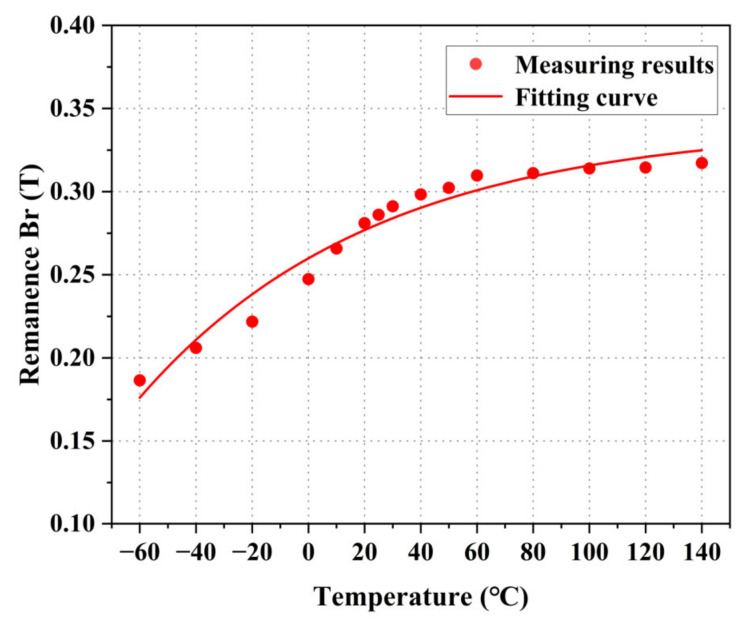
Measurement results of DC remanence $B_{r}$ at different temperatures.

According to the hysteresis loop, the relationship between the remanence $B_{r}$ and temperature of permalloy is analyzed, as shown in Figure 9. The results show that the remanence improves with the increase in temperature as an exponential function. Compared to room temperature (25 °C), the remanence decreases by 34.81% at −60 °C and increases by 8.93% at 140 °C.

For applications in magnetic shielding devices, shielding of low-frequency magnetic fields is also important, so it is necessary to test the AC magnetic properties of materials. The most important of these is the amplitude permeability $\mu_{a}$ (when the magnetic field intensity changes periodically, the relative permeability is obtained by dividing the peak value of the magnetic flux density by the peak value at the specified amplitude of the magnetic field intensity). Therefore, the amplitude magnetic permeability at different temperatures is tested. During the test, the locked Hm is 0.5 A/m, and the frequency is 1 kHz, 500 Hz, 300 Hz, 200 Hz, 100 Hz, 50 Hz, 20 Hz, 10 Hz, 1 Hz, 0.1 Hz, and 0.01 Hz.

**Figure 10:**
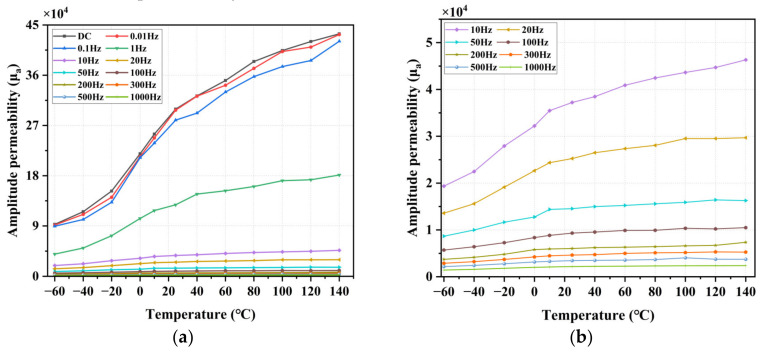
Measurement results of AC amplitude permeability at different temperatures, (**a**) 0.01 Hz to 1 kHz AC permeability at −60 °C to 140 °C, and (**b**) amplification of results from 10 Hz to 1 kHz.

The results of amplitude permeability measured at different ambient temperatures and frequencies are shown in Figure 10. The results show that the amplitude permeability increases with temperature at different frequencies. When the frequency is lower (<0.1 Hz), the amplitude permeability is close to the DC permeability at a magnetic field strength of 0.5 A/m. As the frequency increases, the magnetic permeability of permalloy decreases, and especially when the frequency increases, the correlation between magnetic permeability and temperature decreases, which may be affected by the low-level magnetic permeability at this time.

### 3.3. Discussion

Due to the large difference between the test temperature range and the Curie temperature of the material, the changes in the magnetic properties of permalloy with temperature are not caused by changes in microstructure, which may relate to their intrinsic magnetic changes:

1. The relative permeability μ and coercivity $H_{c}$ of permalloy are closely related to the internal stress of the material. As the temperature increases, the internal stress in the permalloy is gradually released, so the relative magnetic permeability and coercive force of the permalloy gradually decrease with the increase in temperature.
2. The saturation flux density $B_{s}$ is mainly determined by the saturation magnetization strength $M_{s}$. When the temperature is much lower than the Curie temperature of the alloy, the saturation magnetization is similar to the spontaneous magnetization. According to Weiss molecular field theory, a ferromagnet can be equivalent to paramagnetic matter in a huge molecular field, and all atomic magnetic moments tend to be in the same direction, so it can be expressed by the Brillouin function [27,28,29]. Therefore, its saturation magnetization M can be expressed by Equation (8) [30]:
  (8) $$M\left(T\right)=Ng_{J}J\mu_{B}B_{J}\left(y\right)$$
  (9) $$B_{J}\left(y\right)=\frac{2J+1}{2J}\coth\left(\frac{2J+1}{2J}y\right)-\frac{1}{2J}\coth\left(\frac{y}{2J}\right)$$
  (10) $$y=\frac{\mu_{B}B_{J}J\mu_{B}H}{kT}$$
  where N is the number of atoms; g is the Lande factor; J is the total atomic angular momentum; and $\mu_{B}$ is a Bohr magneton.

When the material composition is determined, the variable in the formula is temperature T, and its value gradually decreases as the temperature T increases. Therefore, the saturation flux density $B_{s}$ of permalloy gradually decreases with the increase in temperature.

3. According to the Weiss molecular field theory, with the increase in temperature, the thermal motion in permalloy increases, which makes the atomic magnetic distances tend to be disordered, leading to an increase in the remanence $B_{r}$ in permalloy.
4. In [19], the relative permeability of the electrical steel at 30 °C and 128 °C was analyzed, which increased with increasing temperature. In [20], the relationship between the magnetic properties of ferrite (N87), nanocrystalline (1k107B), and amorphous alloy (1k101) at temperatures ranging from 25 °C to 150 °C was analyzed. The saturation flux density of the three materials decreased with increasing temperature, while the coercivity of nanocrystalline (1k107B) first decreased and then increased with increasing temperature. In [23], the coercivity and saturation flux density of NiFe_2_O_4_ ferrite material were analyzed at 27 °C to 150 °C, both of which decreased with increasing temperature. The relationship between the relative permeability, coercivity, and saturation flux density of magnetic materials and temperature is similar to that of this study, proving the correctness of the discussion.

## 4. Conclusions

In this paper, a magnetic property measurement system for ring-shaped samples of permalloy at different temperatures has been established to measure the magnetic properties of DC and AC (0.01 Hz to 1 kHz) at different temperatures (−60 °C to 140 °C). According to the measurement results, compared with room temperature (25 °C), the magnetic properties of permalloy can be concluded as follows:

- The initial permeability ($\mu_{i}$) improves with the increase in temperature as an exponential function, which decreases by 69.64% at −60 °C and increases by 38.23% at 140 °C.
- The maximum permeability ($\mu_{m}$) improves with the increase in temperature as an exponential function, which decreases by 38.23% at −60 °C and increases by 25.39% at 140 °C.
- The saturation flux density ($B_{s}$) recedes with the increase in temperature as a linear function, which increases by 5.87% at −60 °C and decreases by 11.01% at 140 °C.
- The remanence ($B_{r}$) improves with the increase in temperature as a function of the second-order exponential decay function, which increases by 84.64% at −60 °C and decreases by 22.91% at 140 °C.
- The coercivity ($H_{c}$) recedes with the increase in temperature as an exponential function, which decreases by 34.81% at −60 °C and increases by 8.93% at 140 °C.
- The AC amplitude permeability ($\mu_{a}$) improves with the increase in temperature; it decreases by 69.2% at −60 °C and increases by 45.51% at 140 °C at a frequency of 0.01 Hz, and it decreased by 12.27% at −60 °C and increased by 9.37% at 140 °C at a frequency of 1 kHz. It proves that compared with a higher frequency, the $\mu_{a}$ becomes sensitive to temperature changes at a lower frequency.

According to the measurement and analysis results, temperature has a significant impact on the magnetic properties of permalloy. The measurement results can be introduced into the magnetic analysis of magnetic shielding devices to improve the accuracy of residual magnetic analysis.

## Figures and Tables

**Table 1:** Parameters of the tested standard ring specimen.

Symbol	Parameters	Value
*A*	Outer diameter	40 mm
*B*	Inner diameter	32 mm
*C*	Thickness	5 mm
*S*_x_	Lamination coefficient	99
*L*_e_	Effective magnetic circuit length	112.16 mm
*A*_e_	Effective cross-sectional area	19.52 mm^2^
*N*_1_	Turns of the excitation coil	40
*N*_2_	Turns of the induction coil	20

## Data Availability

Not applicable.
